# Medical educators’ perspectives of teaching physical examinations using ultrasonography at the undergraduate level

**Published:** 2013-03-31

**Authors:** Irene Ma, Ian Wishart, Malgorzata Kaminska, Kevin McLaughlin, Sarah Weeks, David Lautner, Heather Baxter, Bruce Wright

**Affiliations:** 1 Department of Medicine, University of Calgary, Calgary, Alberta, Canada; 2 Department of Emergency Medicine, University of Calgary, Calgary, Alberta, Canada; 3 Department of Family Medicine, University of Calgary, Calgary, Alberta, Canada; 4 Department of Cardiac Sciences, University of Calgary, Calgary, Alberta, Canada; 5 Department of Radiology, University of Calgary, Calgary, Alberta, Canada

## Abstract

**Background:**

Ultrasonography is increasingly used for teaching physical examination in medical schools. This study seeks the opinions of educators as to which physical examinations would be most enhanced by the addition of ultrasonography. We also asked when ultrasound-aided physical examination teaching could have deleterious effects if used outside its intended scope.

**Methods:**

All of the educators from the University of Calgary Master Teacher Program were invited to complete a 22-item paper-based survey. Survey items were generated independently by two investigators, with input from an expert panel (*n* = 5).

**Results:**

Of the 36 educators, 27 (75%) completed the survey. Examinations identified to be potentially most useful included: measuring the size of the abdominal aorta, identifying the presence/absence of ascites, identifying the presence/absence of pleural effusions, and measuring the size of the bladder. Examinations thought to be potentially most harmful included: identifying the presence/absence of intrauterine pregnancy, measuring the size of the abdominal aorta, and identifying the presence/absence of pericardial effusion.

**Conclusions:**

Examinations that are potentially the most useful may also be potentially the most harmful. When initiating an ultrasound curriculum for physical examinations, educators should weigh the risks and benefits of examinations chosen.

## Introduction

Physical examination is a cornerstone of clinical practice. 1–3 While competency in physical examination skills is an explicit goal for undergraduate medical education, 4 gaps in these skills amongst trainees remain a concern. 5–7 With the increase in portability and availability of ultrasound equipment over the past ten years, a number of medical schools have turned to the use of ultrasonography to assist in teaching physical examination skills, demonstrating improvement in physical examination competence and confidence. 8–13

Many academic institutions and learners value the early introduction of ultrasonography skills to medical students. 14–16 Indeed, for a number of years, ultrasonography has been touted as the “stethoscope [sonoscope] of the future,” 17–21 capable of impacting clinical decisions after appropriate training in its use as a point-of-care device. 11,22,23 Concerns abound regarding the potential harm that may stem from either improper training or use of this technology beyond its intended scope. 24,25 Although guidelines and standards exist regarding the training in the use of ultrasound as a point-of-care device in medical specialties such as emergency medicine, 26 radiology, 27 echocardiography, 28 and critical care medicine, 29,30 these standards may not always be feasible within the undergraduate medical education curriculum. Proper curriculum development should take into account both the potential benefits conferred and the potential harms that may result from its improper training or use. For a medical school interested in introducing the use of ultrasound in teaching physical examination skills, what may be the potential harms that may arise from the misuse of this technology by its learners? This study seeks to examine the opinions of medical educators as to which ultrasound examinations may be useful for teaching physical examinations and which may be harmful if learners misapply their skills in the clinical arena.

## Methods

All of the medical educators from the University of Calgary Undergraduate Medical Education Master Teacher Program 31 (2010–2011) were invited to complete a voluntary self-administered 22-item paper-based survey. The majority of educators in this program are generalist physicians who were selected into the program on the basis of a demonstrated track record of providing excellence in education and having a proven interest in teaching medical students. Only consenting educators are included in this study. This study was approved by the University of Calgary Conjoint Health Research Ethics Board.

### Survey Development

Survey domains and a blueprint of survey items were generated independently by two investigators (IM, IW) based on a review of the literature on ultrasound training specific for teaching physical examinations for medical students. 8–12, 14–16,22,23,32–35 Key domains included educator experience, interest in ultrasound, and physical examinations that may be introduced into the undergraduate medical curriculum. Starting with an initial list of 21 examinations, informal feedback on survey items and domains was then obtained from an expert panel (*n* = 5). This panel consisted of a radiologist, a cardiologist specializing in echocardiography, an emergency physician trained in emergency ultrasound, 36,37 a medical educator with experience in curriculum design and implementation 38,39 and over 10 years experience in teaching physical examinations, and a general surgeon with more than 10 years experience in teaching the use of ultrasound for Focused Assessment with Sonography for Trauma (FAST). 40 Informal feedback resulted in a final 16 physical examinations for inclusion into the survey (Appendix A). Examinations of the gallbladder, bowel, cardiac views, ovary and appendix were removed based on the reasons of limited relevance, utility or feasibility to teaching physical examination skills.

### Statistical Analysis

Data were analyzed using descriptive statistics. Comparisons between groups were made with the use of Wilcoxon rank-sum tests and Fisher exact tests. All analyses were performed using STATA 11.2 (StataCorp, College Station, Tx, USA).

## Results

Of the 36 medical educators, 27 (75%) consented and completed the survey. Table 1 lists the demographic characteristics of the medical educators. On average, the medical educators have been in medical practice for 17 (SD = 11) years. The majority of the educators are family medicine practitioners (*n* = 13; 48%) and internists (*n* = 7; 26%). Most do not currently use ultrasound in their practice (*n* = 22; 81%) and report low competency it its use (median score 2, Inter-quartile range (IQR) 1–3; where 1 = very incompetent; 5 = very competent). However, median interest in attending ultrasound training for medical educators was high (5, IQR 2–5, where 1 = very uninterested and 5 = very interested). Those in family medicine expressed higher interest in pursuing further ultrasound training (median interest level 5, IQR 5–5) than those not in family medicine (median 3.5, IQR 1–5; *p* = 0.03).

**Figure 1:**
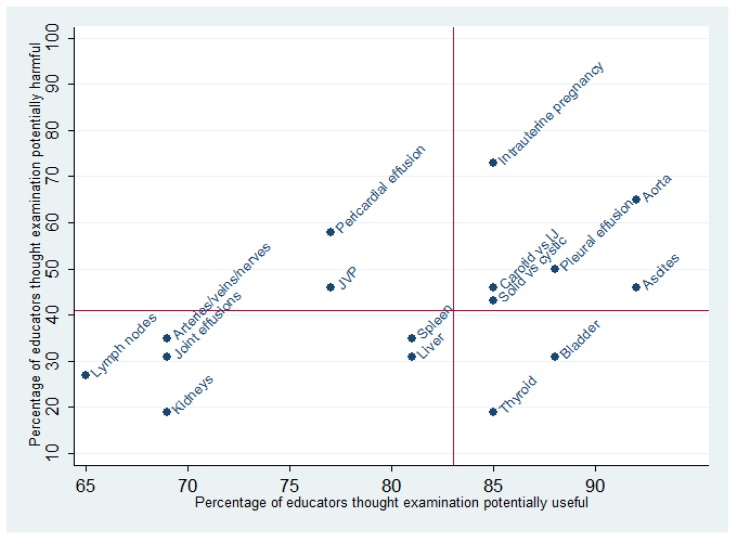
Scatterplot of ultrasound examinations thought to be potentially harmful if misdiagnosed vs. potentially useful for teaching. Red lines shown are median percentages

Of each of the physical examinations listed, at least 65% of educators felt that ultrasonography is potentially useful for teaching. Examinations identified to be potentially **most useful** for teaching physical examinations included: measuring the size of the abdominal aorta (*n* = 24; 92%), identifying the presence/absence of ascites (*n* = 24; 92%), identifying the presence/absence of pleural effusions (*n* = 23; 88%), and measuring the size of the bladder (*n* = 23; 88%) (Table 2 and Figure 1). Examinations identified to be potentially **least useful** for teaching physical examinations included: lymph node examination (*n* = 17; 65%), identifying the location of peripheral arteries/veins/nerves (*n* = 18; 69%), identifying the presence/absence of kidneys (*n* = 18; 69%), and identifying the presence/absence of joint effusions (*n* = 18; 69%).

Examinations thought **most likely to be potentially harmful** if trainees misapply ultrasonography skills included: identifying the presence/absence of intrauterine pregnancy (*n* = 19; 73%), measuring the size of the abdominal aorta (*n* = 17; 65%), and identifying the presence/absence of pericardial effusion (*n* = 15; 58%). Examinations thought **least likely to be potentially harmful** if trainees misapply ultrasonography skills included: thyroid examination (*n* = 5; 19%), identifying the presence/absence of kidneys (*n* = 5; 19%), lymph node examination (*n* = 7; 27%), identifying liver span/location (*n* = 8; 31%), identifying the presence/absence of joint effusions (*n* = 8; 31%), and measuring the size of the bladder (*n* = 8; 31%). Proportions of examinations thought to be useful or harmful did not differ between educators in family medicine and those in other specialties (*p* > 0.05 in all cases).

## Discussion

Our study indicates that, although for a number of examinations the use of ultrasound was thought to be potentially useful for teaching physical examinations, some of these same examinations were also thought to be potentially harmful if findings are misdiagnosed or misinterpreted by the trainee at the bedside. For example, for the measurement of the size of the abdominal aorta and identifying the presence/absence of intrauterine pregnancy, although more than 80% of the educators thought these examinations may be potentially useful for teaching physical examinations, more than 65% of the educators also thought that these examinations may be potentially harmful. Indeed, although the ability to scan the abdominal aorta for size and location may assist in helping trainees recognize proper location for hand placements for feeling for abdominal pulsations, extension of these preliminary skills into making clinical diagnoses may be problematic. For example, a false negative in the identification of an abdominal aortic aneurysm may result in the under-estimation of the risk of rupture while a false positive identification of an abdominal aortic aneurysm may result in unnecessary surgery that is associated with a mortality rate of 4.2% and a complication rate of 32.4%. 41 In a similar vein, a misdiagnosis of a pseudogestational sac as a gestational sac will give false reassurance of an intrauterine pregnancy, whereas a misdiagnosis of an ectopic pregnancy may result in mismanagement of the patient. 42 Thus caution should be exercised in introducing examinations where the potential harm for misdiagnoses is high if the use of ultrasound skills is applied beyond its initial scope.

Of the examinations surveyed, thyroid examination and measuring the size of the bladder by ultrasound were thought to be both potentially useful for teaching physical examinations and unlikely to be harmful. In the selection of curriculum content, we recommend the consideration of examinations in the right lower quadrant of figure 1 (most useful and least harmful) and left lower quadrant (less useful but least harmful).

Our study has a number of limitations. First, this study is a single-center study. Our educators are predominantly generalist physicians. Therefore, generalizability of these results to a different group of educators is unknown. Second, these survey results reflect only the opinions of the educators, who although skilled in physical examination teaching, are not skilled in the use of ultrasound. Examinations thought to be harmful may not in reality pose harm to patients, if proper training and application of point-of care skills are undertaken. Likewise, examinations thought to be potentially useful for training physical examination skills may not in reality provide utility in improving clinical skills. Centers choosing to carefully design educational and assessment activities around what were thought by participants in this study to be potentially harmful examinations should not be discouraged to do so on the basis of this study alone. Future study should survey also the opinions of diagnostic imaging specialists as well as experts in point-of-care ultrasound. Third, our results do not take into account learners’ own ability to recognize their skill limitations. However, given that a number of reports have previously suggested that physicians’ self-assessment abilities may be limited, 43–46 it is reasonable to focus curricular efforts on skills that are less likely to pose harm to patients. Fourth, enthusiasm for ultrasound teaching was noted to be high, which may lead to a trend towards an inflated estimation of utility. Further, our educators have low self-reported competency in ultrasound, which in turn may serve to inflate perceived risks as well. Thus, perceived risks and benefits will need to take into account characteristics of the teacher population. Nonetheless, in the design of a curriculum incorporating new technology and skills to educators unfamiliar with the technology, it is helpful to start with introducing skills that resonate with teachers and learners alike. Specifically, introducing skills that are considered to be the most useful and least harmful is a logical starting point. In an age where technology is increasingly introduced into education, 47,48 careful introduction of technology will optimize faculty buy-in, which is an important element in the success of a new curriculum. 49 Finally, the results of this survey serve only as one step in determining what is or may be an appropriate curriculum for medical students. In devising an ultrasound curriculum, educators need to take into account additional factors such as needs assessments, costs and other feasibility issues.

In conclusion, in devising a physical examination curriculum using ultrasound, there is confusion and disagreement amongst educators on which physical examinations should be integrated with ultrasound training. Physical examinations that are thought to be potentially the most useful to teach with ultrasound may also be potentially the most harmful to the patient if skills are misused by the trainees. Educators need to weigh the risks and benefits of the examinations chosen.

## Figures and Tables

**Table 1:** Demographic Characteristics of Master Teachers (*n* = 27)

Demographic Characteristics	*n* =27
Male sex	13 (48%)
Mean no. of years in practice (SD)*	17 (11)
Primary area of specialty	
Emergency Medicine	1 (4%)
Family Medicine	13 (48%)
Internal Medicine	7 (26%)
Obstetrics and gynecology	1 (4%)
Pediatrics	2 (7%)
Surgery	3 (11%)
Currently uses ultrasound in practice	5 (19%)
Median self-reported competency in ultrasound**	2 (IQR 1–3; range 1–5)

* SD denotes standard deviation

** This variable was coded on a 5-point Likert scale (1 = Very incompetent; 5 = Very competent)

**Table 2:** Perceived Utility of Using Ultrasound in Teaching Physical Examinations (*n* = 27)*

Examination	Thought to be potentially useful for teaching physical examination *n* (%)	Thought to be potentially harmful if ultrasonography skills are misapplied *n* (%)
Liver (span/location)	21 (81)	8 (31)
Spleen (span/location)	21 (81)	9 (35)
Lymph nodes (size/location)	17 (65)	7 (27)
Thyroid gland	22 (85)	5 (19)
Jugular venous pressure	20 (77)	12 (46)
Location of peripheral arteries/veins/nerves	18 (69)	9 (35)
Presence or absence of:		
Kidneys	18 (69)	5 (19)
Joint effusions	18 (69)	8 (31)
Ascites	24 (92)	12 (46)
Pleural effusion	23 (88)	13 (50)
Pericardial effusion	20 (77)	15 (58)
Intrauterine pregnancy	22 (85)	19 (73)
Differentiating between carotid artery and internal jugular vein	22 (85)	12 (46)
Differentiating between solid and cystic lesions	22 (85)	12 (46)
Measuring the size of:		
Bladder	23 (88)	8 (31)
Abdominal aorta	24 (92)	17 (65)

* Denominator not consistently 27, as not every participant answered every question.
